# PartCrafter: find, generate and analyze BioParts

**DOI:** 10.1093/synbio/ysz014

**Published:** 2019-06-04

**Authors:** Emily Scher, Shay B Cohen, Guido Sanguinetti

**Affiliations:** School of Informatics, University of Edinburgh, Edinburgh, UK

**Keywords:** parts-based design, bioparts, search, data aggregation, design

## Abstract

The field of Synthetic Biology is both practically and philosophically reliant on the idea of BioParts—concrete DNA sequences meant to represent discrete functionalities. While there are a number of software tools which allow users to design complex DNA sequences by stitching together BioParts or genetic features into genetic devices, there is a lack of tools assisting Synthetic Biologists in finding BioParts and in generating new ones. In practice, researchers often find BioParts in an *ad hoc* way. We present PartCrafter, a tool which extracts and aggregates genomic feature data in order to facilitate the search for new BioParts with specific functionalities. PartCrafter can also turn a genomic feature into a BioPart by packaging it according to any manufacturing standard, codon optimizing it for a new host, and removing forbidden sites. PartCrafter is available at partcrafter.com.

## 1. Introduction

Parts-based design has been a central tenet of *Synthetic Biology* since the field’s inception. Endy (1) described how sequences should be designed using an abstraction hierarchy, where devices could be built from parts, and systems could be built from devices. Almost all of the popular DNA design tools for Synthetic Biology are built around the parts-based model, including SnapGene, Genome Compiler and Benchling. These tools provide a library of features or parts—sequences of DNA that encode for a specific biological function (2), sometimes called BioParts. Using these libraries, or parts of their own, users can easily design complex genetic systems.

However, DNA design tools rely on existing part libraries and do not provide an automated way of finding and generating parts. This is not surprising: ‘it is currently easier to assemble multi-part genetic circuits consisting of several BioParts, or even entire genomes, than it is to reliably predict how these BioParts will interact in the final system’ (3). Many existing BioParts rely on disparate pieces of a genome, contextual conditions, and luck for their ‘expected’ functionality to come to light. Unless characterization experiments have been performed for a part in a wide variety of circumstances, it is impossible to know how the part will behave *in vivo*.

PartCrafter was built to help users make informed decisions about which genomic features would make sensible BioParts for their experiments. We have enabled rational search of genomic features by leveraging existing annotated data from YeastMine (4), SynBioMine, ThaleMine (5), The Saccharomyces Genome Database (6), UniProt (7), various NCBI databases, PubMed and DOOR (8). Unlike other, hand-curated parts libraries, like the Registry of Standard Biological Parts, PartCrafter is not limited to a certain number of organisms, manufacturing standards, or a certain subset of parts, but can handle a theoretically unlimited number and variety of genomic features.

Other tools exist which allow users generate to BioParts, such as J5 (9) and GeneDesign (10). However, these tools require that the user already knows what genomic feature they want to turn into a part. PartCrafter allows users who do not have a genomic feature in mind to find and generate the BioParts that they need. Additionally, unlike other *Synthetic Biology* search tools, PartCrafter does not require the user to provide sequence or annotation data. Our extensive data aggregation allows users to search quickly and easily for features, and to find more illuminating results. The differences between PartCrafter and several other related tools are documented in Table 1.

**Table 1.** Comparison of software tools for finding and generating BioParts

	Unlimited number of parts/features	Part generation	Search capabilities	User must provide the data	Free to use
Parts Registry	Only the 20 000 Parts in the database	Yes	Full search of parts in the database	No	Yes
J5	Yes	Yes	No search capabilities	Yes	Yes
GeneDesign	Yes	Yes	No search capabilities	Yes	Yes
BioPartsBuilder	Yes	Yes	Full-text search and filtering of the GFF file data	No	Yes
Archetype	Yes	No	Full-text search of user-provided data	Yes	No
SynBioHub	Yes	No	Full-text search of user-provided data	No, though all data is user-provided	Yes
PartCrafter	Yes	Yes	Full-text search and filtering of extensive aggregated descriptive data	No	Yes

While many databases allow users to search for sequences using feature identifiers, scientists are hindered by being unable to link these sequences with functional meaning. Synthetic Biologists especially need a tool which can link sequence text with functional characteristics if they are to be able to design complex systems with a reasonable level of accuracy.

## 2. Workflow

**Figure 1.**
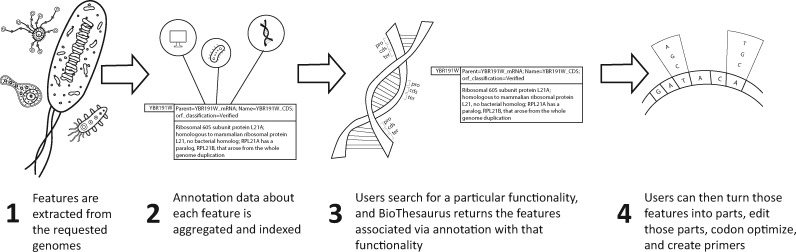
The workflow of PartCrafter.

The PartCrafter workflow consists of four steps: Organism Processing, Data Aggregation, Search and Part Generation. These steps are summarized in Figure 1, and described further below.

### 2.1 Organism processing and data aggregation

PartCrafter can process any genome, but it comes pre-loaded with four model organisms: *Saccharomyces cerevisiae*, *Escherichia coli*, *Arabidopsis thaliana*, and *Schizosaccharomyces pombe*. A genome is added by uploading a standard GFF file containing the genome sequence. When a new genome is uploaded to the application, the application first extracts all of the genome’s features, and then aggregates descriptive data about each of these features. These data come from a number of sources, documented above. The specific fields included are documented in Table 2. PartCrafter then uses this aggregated data to build an index through Lucene (11). To build an index, Lucene first breaks the text up into terms, and then associates each term with the documents which contain it. This inverted index—so called because it is the inverse of the more natural relationship between documents and terms—allows Lucene to quickly return all documents related to the search terms inputted by the user.

**Table 2.** Fields aggregated from data sources

Database	Fields
SynBioMine	Description, feature.description, feature. identifier, feature.name, protein.name
YeastMine	BriefDescription, description, name, phenotype Summary, functionSummary
ThaleMine	BriefDescription, computational Description
NCBI (protein)	Comment, description, keywords
NCBI (nucleotide)	Comment, description, keywords
DOOR	Species, size, synonyms, symbols
PubMed	ArticleTitle, AbstractText

Uploading a new genome involves processing the file, extracting out the annotated features, and, for each of those features, aggregating data from our variety of sources. This involves hundreds of thousands of requests in total, and, because these requests are made with delays in between them to prevent overloading the servers of our data sources, this takes several days for each genome. Because this is a long and computationally intense process taking up significant amounts of memory, only administrative users of PartCrafter can upload new organisms themselves. However, the tool includes a form which allows users to request that a new organism is added.

### 2.2 Search

There are two ways to search for a feature with PartCrafter. First, a user can input a description of the features that they would like. PartCrafter will then output the features in the database whose aggregated texts best match the requested description. In this way, users can find parts associated with a particular functionality. Users can also filter their searches using tags. For example, to search for all features related to cell death, a user would simply search for ‘cell death.’ However, to search for all features related to cell death that occur in *S. cerevisiae*, they would use the following query.

‘cell death’ AND organism_name:‘*Saccharomyces cerevisiae*’

The tags which can be used with PartCrafter are documented fully on the website. This particular query has 42 results, the top 10 of which are summarized in Table 3.

**Table 3.** Summary of the top results for the query provided in Section 2.2

Result number	Systematic name	Feature name	Summary of the descriptive data
1	YNR074C	AIF1	Mitochondrial cell death effector
2	YGL203C	KEX1	Cell death protease essential for hypochlorite-induced apoptosis
3	YNL305C	BXI1	Protein involved in apoptosis
4	YLR011W	LOT6	Flavin mononucleotide (FMN)-dependent NAD(P)H:quinone reductase. Role in apoptosis-like cell death.
5	YMR074C	SDD2	Protein with homology to human PDCD5. PDCD5 is involved in programmed cell death.
6	YGL231C	EMC4	Member of conserved ER transmembrane complex.
7	YHR179W	OYE2	Conserved NADPH oxidoreductase containing FMN. May be involved in sterol metabolism, oxidative stress response and programmed cell death.
8	YKL184W	SPE1	Ornithine decarboxylase. Deletion decreases lifespan, and increases necrotic cell death and ROS generation.
9	YPL171C	OYE3	Conserved NADPH oxidoreductase containing FMN. Has potential roles in oxidative stress response and programmed cell death.
10	YKR042W	UTH1	Mitochondrial inner membrane protein. Implicated in cell wall biogenesis, the oxidative stress response, life span during starvation, and cell death.

Additionally, users can search for features which are maximally similar to a feature of interest. The user inputs the name of their feature of interest, and PartCrafter outputs the features whose descriptive data are the most similar to the descriptive data of the specified feature.

All search results are based entirely on annotation data. This is largely because experimental data are incredibly sparse. However, previously, this annotation data have been disparate, and impossible to search centrally (12).

The search functionality was built using the search engine library Lucene (11). The ‘Search by Function’ feature uses full-text search to find matches to the query string. The ‘Find Similar Features’ feature uses the Lucene ‘More Like This’ query, which searches for documents most similar to a selected document of interest.

### 2.3 Parts generation

Once the user has found their feature or features of interest, PartCrafter allows them to generate the specific BioParts they need.

PartCrafter extracts the feature sequence from its genome, along with its promoter and terminator sequences, if applicable, as specified by the user. Then, the user is able to specify the manufacturing standard they would like to use to package their part, or create their own. The user can then search for forbidden restriction sites, and add their manufacturing standard’s required overhangs. However, finding forbidden sites does not automatically remove them—the user can choose to do so by codon optimizing their part to remove the sites. Additionally, they can codon optimize the part for any host. If the user already has the strain the feature comes from in their laboratory, there is no need for them to synthesize the sequences *de novo*. Instead, they can use the primer generation form to generate primer sequences which will allow them to PCR the sequences out of the host genome. This functionality is all based on that of Genome Carver (13). The primers are generated using Primer3 (14). The codon optimization is done using DNAChisel (15). The DnaChisel codon optimization algorithm uses a dynamic programming approach and codon usage tables for each organism to build sequences which meet desired constraints. These constraints can be, for example, to optimize the sequence for a particular organism, or to remove unwanted sequences, such as restriction sites.

Finally, the user can download their parts in CSV format using the download button. Currently, only CSV format is supported.

## 3. Example use case

We illustrate the use of PartCrafter in a simple and generic scenario.

A researcher is investigating programmed cell death in *S. cerevisiae*. In order to design synthetic DNA circuits using a common DNA design software tool, they first need to find genes related to cell death, and turn them into BioParts.

First, the researcher navigates to **partcrafter.com**, and then to the ‘Find Features’ section. The search for features using the following query:

‘cell death’ AND organism_name:‘*Saccharomyces cerevisiae*’

This query searches for all features in the database related to cell death, limiting the results to those in *S. cerevisiae*.

PartCrafter now displays the results, including several genes the researcher would like to turn into BioParts. One such gene is YMR074C, a homolog of Human PDCD5 protein which promotes programmed cell death. The researcher turns this feature into a BioPart by pressing the ‘Make into a Part’ button, which brings up the ‘Generate a Part’ form. This form pulls out the relevant feature sequence, along with the promoter and terminator sequences. The researcher then edits the sequences, adding the relevant manufacturing standard overhangs to the transcriptional unit, and removing forbidden sites.

As the researcher would like to generate a number of parts, they then navigate to the ‘Bulk Query’ tab of the ‘Generate Parts’ screen. There, they are able to generate and edit several parts at once.

The researcher realizes that they do not need to synthesize all of the features. They have *S. cerevisiae* in their lab strain collection, and can generate some of their required sequences through PCR. For these features, the researcher generates cloning primers using the Primer Generation form.

Without PartCrafter, this simple pipeline would have required several different databases and tools. For example, to find the features of interest, the researcher would have potentially had to search SGD, NCBI, and Yeastmine. Once they found their list of features, they would have had to add the overhangs by hand, or turned to one of several part generation tools, for example GeneDesign or J5.

In contrast, PartCrafter is a one stop shop. It is possible to find genomic features which would be useful for an experiment, edit them as necessary to turn them into BioParts, and generate primer sequences which will allow the features to be amplified out of an organism. Further, the final generated sequences can be outputted in CSV format for easy use with part-based design tools.

As shown in Table 1, several tools exist which allow users to search for genomic features, and several tools exist which can turn specific DNA sequences into BioParts. However, PartCrafter is the only data aggregation and search platform built with the specific aim of helping biologists find and build the BioParts that they need for their experiments. PartCrafter offers a streamlined alternative to using a various other disjointed databases and tools, while also providing more illuminating search results.

## 4. Validation

The validation of our tool was two-pronged.

First, we held a workshop at the UK Centre for Mammalian Synthetic Biology Research, an EPSRC funded center at the University of Edinburgh. Each of our participants were researchers—PhD students and postdocs—in a Synthetic Biology lab at the University of Edinburgh, and were familiar with popular DNA design tools.

Each participant was asked to choose any *E. coli* or *S. cerevisiae* gene, and write a short description of its function. They were then asked to search PartCrafter using their short description. For each of these searches, the gene of interest occurred in the top two results 5/5 times, and as the top search result 3/5 times.

Each participant was also asked to rate each of the top 10 search results for their query as either ‘not relevant,’ ‘somewhat relevant’ or ‘very relevant.’ Over all of the queries, 86% of the top 10 results were at least somewhat relevant to the query, and 32% of the results were very relevant to the query. Eighty-eight percent of the top five results were at least somewhat relevant, and 40% were very relevant.

The worksheet used in this workshop is available on the PartCrafter website, under the ‘Help’ section. It provides some quick exercises to help users learn how to use PartCrafter. Users are able to submit their completed worksheets to us, which will help us to continually verify that our search results are of good quality.

Additionally, we programmatically validated our search results by comparing them to another database. WikiGenes (16) is a collaborative database for genetic annotation data. Uniquely for this type of data aggregation, it offers an API, and the data can be edited by anyone, with the intent that researchers will be able to crowdsource their expertise.

In order to validate the PartCrafter search results, we identified 468 genes which have entries in both of these databases. These genes came from *E. coli*, *S. cerevisiae* and *S. pombe*, as WikiGenes does not have entries on genes from *A. thaliana*. For each of these genes, PartCrafter was queried using the data from the WikiGenes entry as the query string. Our search results were then scored using the mean reciprocal rank, a standard metric to evaluate information retrieval systems. This gave us a score of approximately 0.569, indicating that, on average, the correct gene was listed second in the PartCrafter search results.

Interestingly, there was a significant difference in this figure when looking at genes from the individual organisms. The mean reciprocal rank was 0.668 for just the *S. cerevisiae* genes, 0.410 for the *E. coli* genes, and 0.546 for the *S. pombe* genes. This variability likely speaks to the comparative quality of annotation data for these different organisms, either in the WikiGenes database, PartCrafter database, or both.

In total, the desired result appeared in the top five search results 70.1% of the time. For *E. coli* genes, the desired result was in the top five 48.2% of the time, for *S. cerevisiae* 81.6% of the time, and for *S. pombe* 69.9% of the time.

These metrics do not offer a perfect comparison between the two data sources. For instance, there are many genes listed in PartCrafter which are not in WikiGenes, which may well have affected the search results. The two databases also, of course, have differing annotation data, which means that we cannot expect a reciprocal rank of 1. That being said, these results are quite encouraging, as they demonstrate that, in general, PartCrafter highly ranks relevant entries.

## Availability

PartCrafter is available at *partcrafter.com*. It is not open source, however, docker images of the PartCrafter services are publicly available. Instructions for setting up a PartCrafter server are available on the website. An API is available with instructions for use on the website help page. Additionally, the authors agree to maintain the application for at least 2 years from the data of publication.
